# Supplementary material for: Guanine nucleotide biosynthesis blockade impairs MLL complex formation and sensitizes leukemias to menin inhibition
Source: Nat Commun. 2025 Mar 18;16:2641. doi: 10.1038/s41467-025-57544-9 (PMC11920272; doi:10.1038/s41467-025-57544-9)
Supplement: Supplementary file 1 — Supplementary Information [file 41467_2025_57544_MOESM1_ESM.pdf]

Supplemental information

Guanine nucleotide biosynthesis blockade impairs MLL complex formation and sensitizes leukemias to menin inhibition

Xiangguo Shi, Minhua Li, Zian Liu, Jonathan Tiessen, Yuan Li, Jing Zhou, Yudan Zhu, Swetha Mahesula, Qing Ding, Lin Tan, Mengdie Feng, Yuki Kageyama, Yusuke Hara, Jacob J. Tao, Xuan Luo, Kathryn A. Patras, Philip L. Lorenzi, Suming Huang, Alexandra M. Stevens, Koichi Takahashi, Ghayas C. Issa, Md. Abul Hassan Samee, Michalis Agathocleous, and Daisuke Nakada

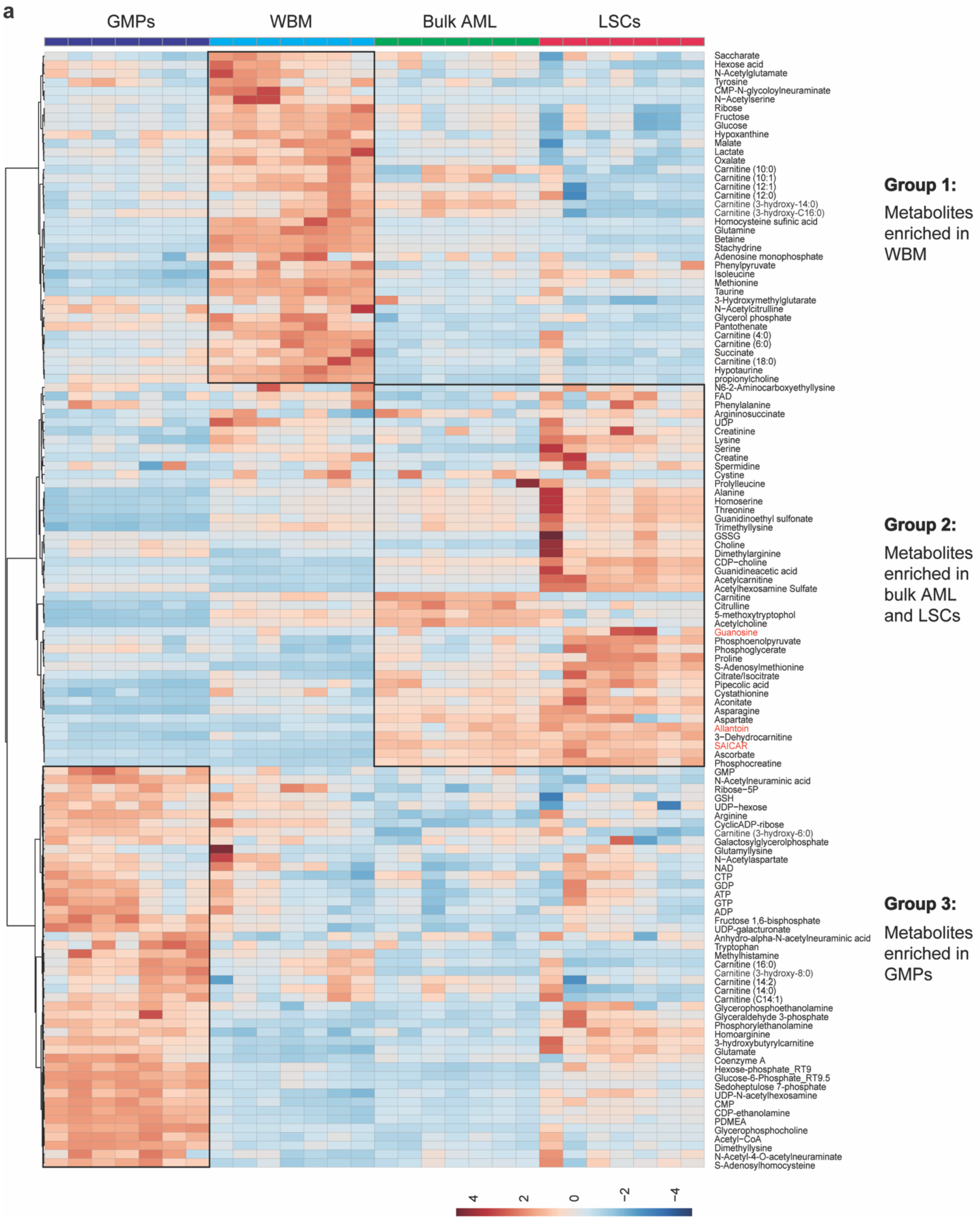

a
GMPs
WBM
Bulk AML
LSCs
Saccharate
Hexose acid
N-Acetylglutamate
Tyrosine
CMP-N-glycoloylneuraminate
N−Acetylserine
Ribose
Fructose
Glucose
Hypoxanthine
Malate
Lactate
Oxalate
Carnitine (10:0)
Carnitine (10:1)
Carnitine (12:1)
Carnitine (12:0)
Carnitine (3-hydroxy-14:0)
Carnitine (3-hydroxy-C16:0)
Homocysteine sufinic acid
Glutamine
Betaine
Stachydrine
Adenosine monophosphate
Phenylpyruvate
Isoleucine
Methionine
Taurine
3-Hydroxymethylglutarate
N−Acetylcitrulline
Glycerol phosphate
Pantothenate
Carnitine (4:0)
Carnitine (6:0)
Succinate
Carnitine (18:0)
Hypotaurine
propionylcholine
N6-2-Aminocarboxyethyllysine
FAD
Phenylalanine
Argininosuccinate
UDP
Creatinine
Lysine
Serine
Creatine
Spermidine
Cystine
Prolylleucine
Alanine
Homoserine
Threonine
Guanidinoethyl sulfonate
Trimethyllysine
GSSG
Choline
Dimethylarginine
CDP−choline
Guanidineacetic acid
Acetylcarnitine
Acetylhexosamine Sulfate
Carnitine
Citrulline
5-methoxytryptophol
Acetylcholine
Guanosine
Phosphoenolpyruvate
Phosphoglycerate
Proline
S-Adenosylmethionine
Citrate/Isocitrate
Pipecolic acid
Cystathionine
Aconitate
Asparagine
Aspartate
Allantoin
3−Dehydrocarnitine
SAICAR
Ascorbate
Phosphocreatine
GMP
N-Acetylneuraminic acid
Ribose−5P
GSH
UDP−hexose
Arginine
CyclicADP-ribose
Carnitine (3-hydroxy-6:0)
Galactosylglycerolphosphate
Glutamyllysine
N−Acetylaspartate
NAD
CTP
GDP
ATP
GTP
ADP
Fructose 1,6-bisphosphate
UDP-galacturonate
Anhydro-alpha-N-acetylneuraminic acid
Tryptophan
Methylhistamine
Carnitine (16:0)
Carnitine (3-hydroxy-8:0)
Carnitine (14:2)
Carnitine (14:0)
Carnitine (C14:1)
Glycerophosphoethanolamine
Glyceraldehyde 3-phosphate
Phosphorylethanolamine
Homoarginine
3-hydroxybutyrylcarnitine
Glutamate
Coenzyme A
Hexose-phosphate_RT9
Glucose-6-Phosphate_RT9.5
Sedoheptulose 7-phosphate
UDP-N-acetylhexosamine
CMP
CDP-ethanolamine
PDMEA
Glycerophosphocholine
Acetyl−CoA
Dimethyllysine
N-Acetyl-4-O-acetylneuraminate
S-Adenosylhomocysteine
Group 1:
Metabolites enriched in WBM
Group 2:
Metabolites enriched in bulk AML and LSCs
Group 3:
Metabolites enriched in GMPs
4
2
0
−2
−4

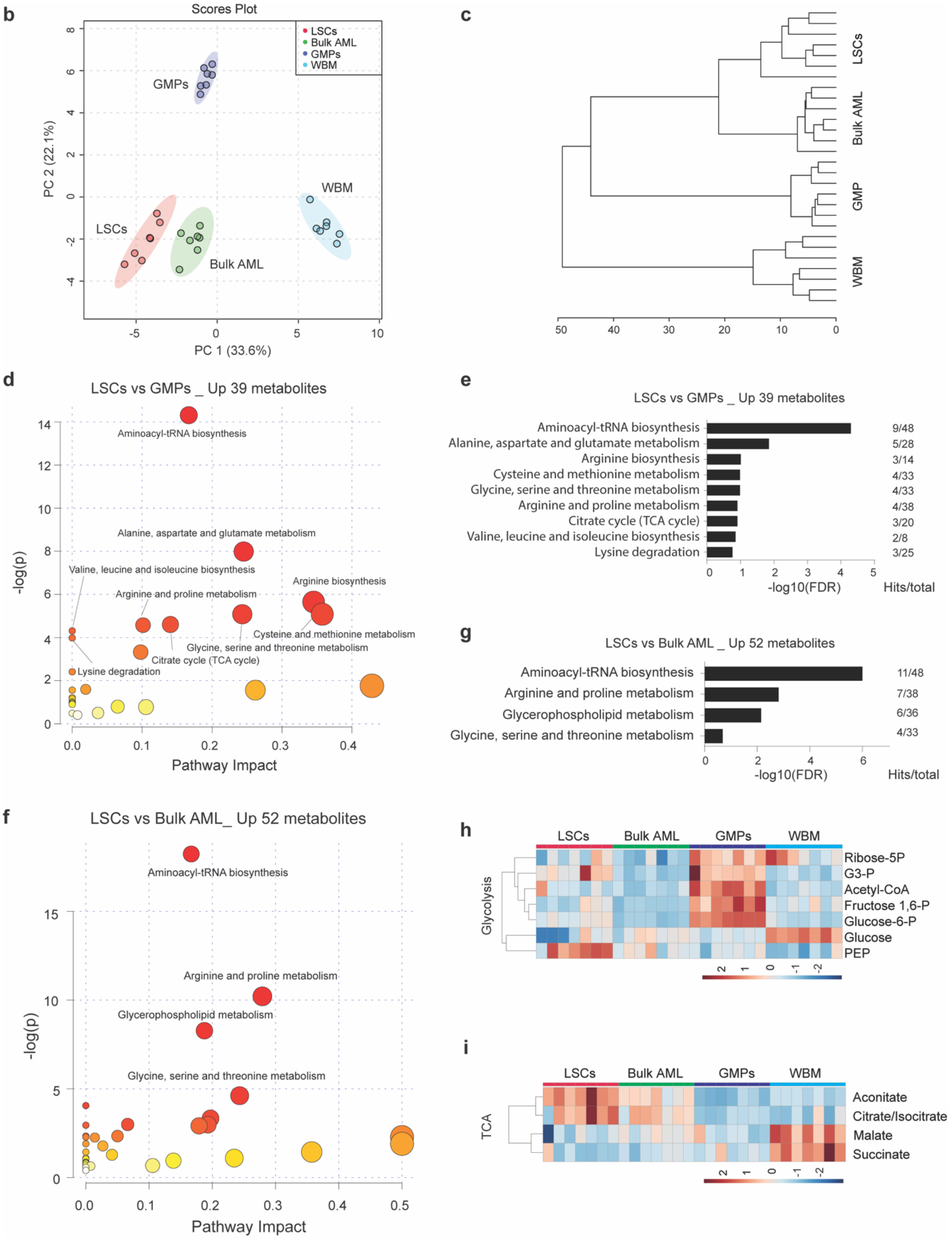

b
Scores Plot
LSCs
Bulk AML
GMPs
WBM
PC 1 (33.6%)
PC 2 (22.1%)
c
LSCs
Bulk AML
GMP
WBM
d
LSCs vs GMPs _ Up 39 metabolites
Aminoacyl-tRNA biosynthesis
Alanine, aspartate and glutamate metabolism
Valine, leucine and isoleucine biosynthesis
Arginine and proline metabolism
Arginine biosynthesis
Cysteine and methionine metabolism
Glycine, serine and threonine metabolism
Citrate cycle (TCA cycle)
Lysine degradation
Pathway Impact
-log(p)
e
LSCs vs GMPs _ Up 39 metabolites
Aminoacyl-tRNA biosynthesis 9/48
Alanine, aspartate and glutamate metabolism 5/28
Arginine biosynthesis 3/14
Cysteine and methionine metabolism 4/33
Glycine, serine and threonine metabolism 4/33
Arginine and proline metabolism 4/38
Citrate cycle (TCA cycle) 3/20
Valine, leucine and isoleucine biosynthesis 2/8
Lysine degradation 3/25
-log10(FDR)
Hits/total
g
LSCs vs Bulk AML _ Up 52 metabolites
Aminoacyl-tRNA biosynthesis 11/48
Arginine and proline metabolism 7/38
Glycerophospholipid metabolism 6/36
Glycine, serine and threonine metabolism 4/33
-log10(FDR)
Hits/total
f
LSCs vs Bulk AML_ Up 52 metabolites
Aminoacyl-tRNA biosynthesis
Arginine and proline metabolism
Glycerophospholipid metabolism
Glycine, serine and threonine metabolism
Pathway Impact
-log(p)
h
LSCs
Bulk AML
GMPs
WBM
Glycolysis
Ribose-5P
G3-P
Acetyl-CoA
Fructose 1,6-P
Glucose-6-P
Glucose
PEP
i
LSCs
Bulk AML
GMPs
WBM
TCA
Aconitate
Citrate/Isocitrate
Malate
Succinate

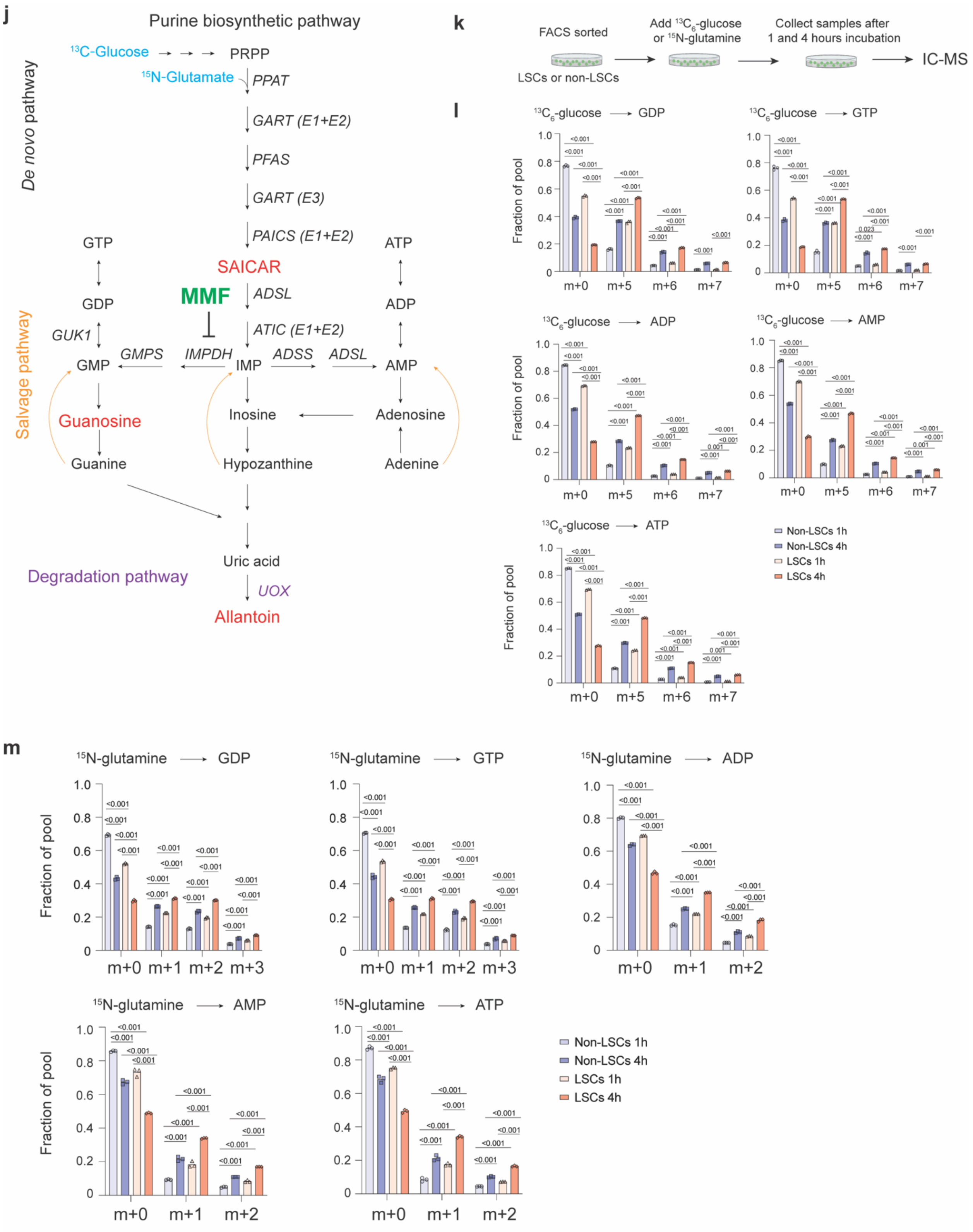

Supplementary Fig. 1. Targeted metabolomics in normal hematopoietic cells and MLL-AF9-driven AML cells, related to Fig. 1.

(**a**) Heatmap of the metabolites in LSCs, bulk AML, GMPs, and WBM cells. These metabolites were classified into 3 groups: group 1, metabolites enriched in WBM cells; group 2, metabolites enriched in bulk AML and LSCs; and group 3, metabolites enriched in GMPs (n=7).

(**b**-**c**) Principal component analysis (PCA) plot (**b**) and dendrogram (**c**) of metabolites in LSCs, bulk AML, GMPs, and WBM cells (n=7).

(**d**-**e**) Metabolic pathway analysis plot (**d**) and pathway analysis (**e**) of 39 upregulated metabolites in LSCs compared to GMPs. The top metabolic pathways are shown based on false discovery rate (FDR) ≤ 0.25 and $p \leq 0.05$.

(**f**-**g**) Metabolic pathway analysis plot (**f**) and pathway analysis (**g**) of 52 upregulated metabolites in LSCs compared to bulk AML cells. The top metabolic pathways are shown based on FDR ≤ 0.25 and $p \leq 0.05$.

(**h**-**i**) Heatmap depicting metabolites involved in glycolysis (**h**) and tricarboxylic acid (TCA) cycle (**i**) in LSCs, bulk AML, GMPs, and WBM cells (n=7).

(**j**) Schematic representation depicting the purine biosynthetic pathway, including the *de novo*, salvage, and degradation pathways. The metabolites in red indicate those enriched in LSCs. $^{13}C_6$-glucose and amide-^{15}N-glutamine (in blue) can be incorporated into the purine biosynthetic pathway.

(**k**) Schematic flowchart depicting the isotype tracing experiments using $^{13}C_6$-glucose or amide-^{15}N-glutamine in LSCs.

(**l**-**m**) Fractional labeling of GDP, GTP, ADP, AMP, and ATP in LSCs and non-LSCs treated with $^{13}C_6$-glucose (**l**) or amide-^{15}N-glutamine (**m**) for 1 and 4 hours (n=3). h, hour.

All data are presented as mean ± SD. p values in this figure were calculated by ANOVA with multiple comparisons analysis using Bonferroni correction post hoc analyses (**l, m**). Source data are provided as a Source Data file.

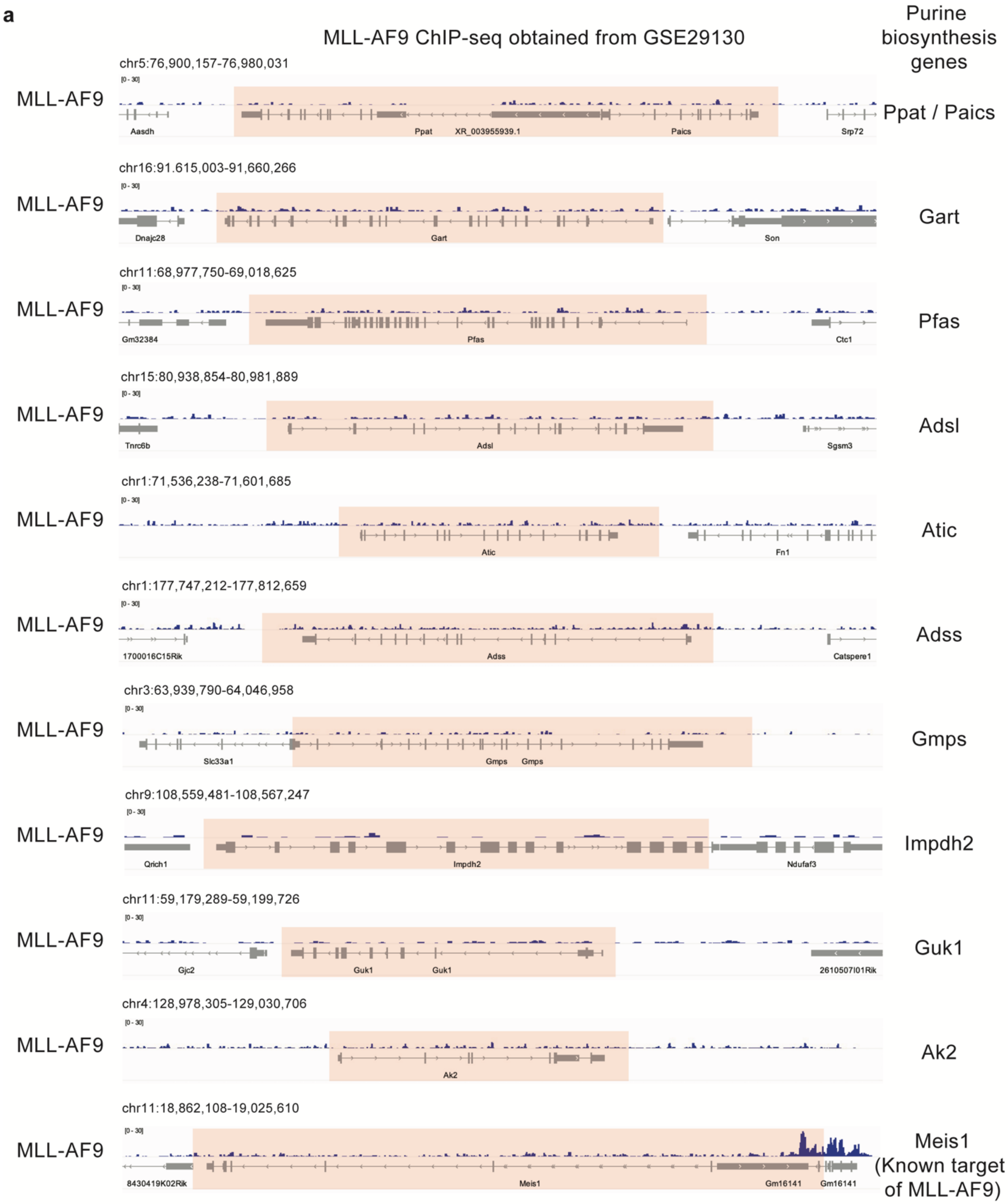
a
MLL-AF9 ChIP-seq obtained from GSE29130
Purine biosynthesis genes
chr5:76,900,157-76,980,031
MLL-AF9
Aasdh
Ppat
XR_003955939.1
Paics
Srp72
Ppat / Paics
chr16:91,615,003-91,660,266
MLL-AF9
Dnajc28
Gart
Son
Gart
chr11:68,977,750-69,018,625
MLL-AF9
Gm32384
Pfas
Ctc1
Pfas
chr15:80,938,854-80,981,889
MLL-AF9
Tnrc6b
Adsl
Sgsm3
Adsl
chr1:71,536,238-71,601,685
MLL-AF9
Atic
Fn1
Atic
chr1:177,747,212-177,812,659
MLL-AF9
1700016C15Rik
Adss
Catspere1
Adss
chr3:63,939,790-64,046,958
MLL-AF9
Slc33a1
Gmps
Gmps
Gmps
chr9:108,559,481-108,567,247
MLL-AF9
Qrich1
Impdh2
Ndufaf3
Impdh2
chr11:59,179,289-59,199,726
MLL-AF9
Gjc2
Guk1
Guk1
2610507I01Rik
Guk1
chr4:128,978,305-129,030,706
MLL-AF9
Ak2
Ak2
chr11:18,862,108-19,025,610
MLL-AF9
8430419K02Rik
Meis1
Gm16141
Gm16141
Meis1 (Known target of MLL-AF9)

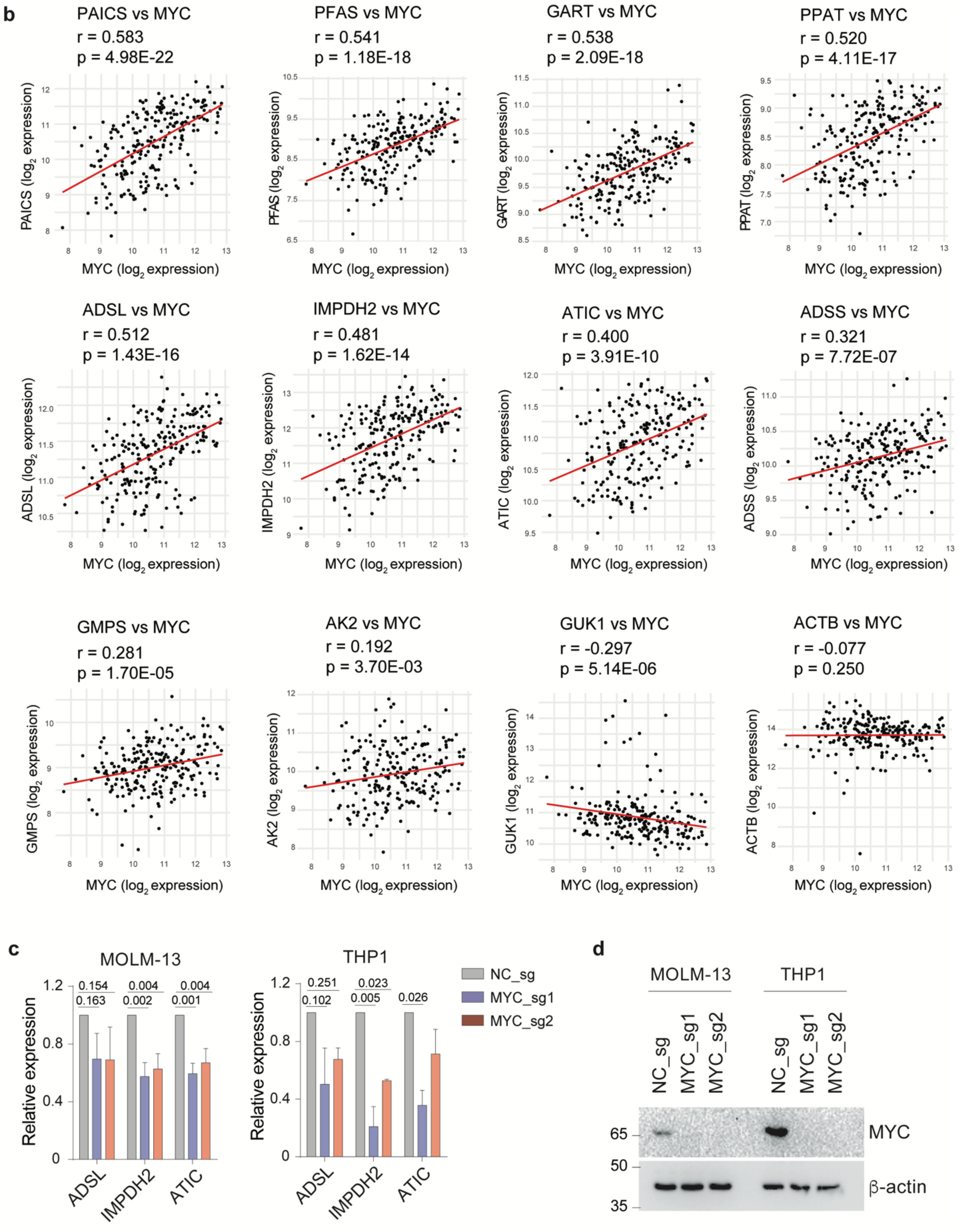
b
PAICS vs MYC
r = 0.583
p = 4.98E-22
PAICS (log2 expression)
MYC (log2 expression)
PFAS vs MYC
r = 0.541
p = 1.18E-18
PFAS (log2 expression)
MYC (log2 expression)
GART vs MYC
r = 0.538
p = 2.09E-18
GART (log2 expression)
MYC (log2 expression)
PPAT vs MYC
r = 0.520
p = 4.11E-17
PPAT (log2 expression)
MYC (log2 expression)
ADSL vs MYC
r = 0.512
p = 1.43E-16
ADSL (log2 expression)
MYC (log2 expression)
IMPDH2 vs MYC
r = 0.481
p = 1.62E-14
IMPDH2 (log2 expression)
MYC (log2 expression)
ATIC vs MYC
r = 0.400
p = 3.91E-10
ATIC (log2 expression)
MYC (log2 expression)
ADSS vs MYC
r = 0.321
p = 7.72E-07
ADSS (log2 expression)
MYC (log2 expression)
GMPS vs MYC
r = 0.281
p = 1.70E-05
GMPS (log2 expression)
MYC (log2 expression)
AK2 vs MYC
r = 0.192
p = 3.70E-03
AK2 (log2 expression)
MYC (log2 expression)
GUK1 vs MYC
r = -0.297
p = 5.14E-06
GUK1 (log2 expression)
MYC (log2 expression)
ACTB vs MYC
r = -0.077
p = 0.250
ACTB (log2 expression)
MYC (log2 expression)
c
MOLM-13
Relative expression
0.154
0.163
0.004
0.002
0.004
0.001
ADSL
IMPDH2
ATIC
THP1
Relative expression
0.251
0.102
0.023
0.005
0.026
ADSL
IMPDH2
ATIC
NC_sg
MYC_sg1
MYC_sg2
d
MOLM-13
THP1
NC_sg
MYC_sg1
MYC_sg2
NC_sg
MYC_sg1
MYC_sg2
65
MYC
50
β-actin
35

Supplementary Fig. 2. Purine biosynthetic genes are regulated by MYC, related to Fig. 2

(**a**) IGV tracks of the MLL-AF9 ChIP-seq signals (GSM721212 of GSE29130) at *Ppat, Paics, Gart, Pfas, Adsl, Atic, Adss, Gmps, Impdh2, Guk1, Ak2* and *Meis1*. MLL-AF9 binding to *Meis1*, a known gene regulated directly by MLL-AF9, is shown as a positive control.

(**b**) Scatter plots depicting the correlation between *MYC* gene expression and purine biosynthesis genes, alongside the *ACTB* gene as a negative control, using publicly available datasets in human AML cells (GSE76009), related to Figure 2D (n=227). r, correlation coefficient.

(**c**-**d**) Relative expression of purine biosynthetic genes (**c**) and immunoblots of MYC and b-actin protein expression on separate membranes (**d**) in MOLM-13 and THP-1 cells expressing sgRNAs targeting control or *Myc* (n=3). NC, negative control.

All data are presented as mean ± SD. p values in this figure were calculated by Pearson/Spearman correlation test (**b**) or ANOVA with multiple comparisons analysis using Dunnett's post hoc analyses (**c**). Source data are provided as a Source Data file.

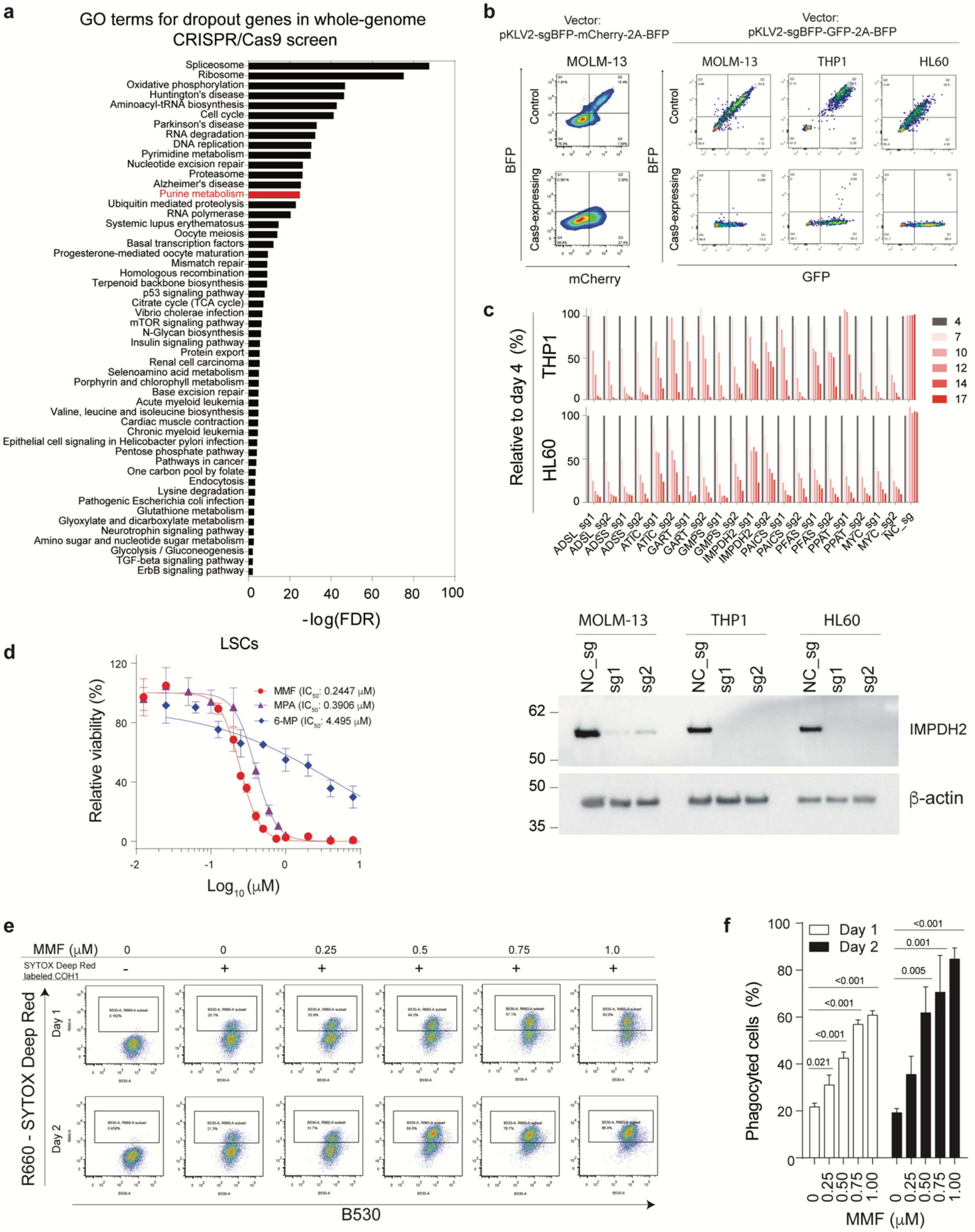
a
GO terms for dropout genes in whole-genome CRISPR/Cas9 screen
Spliceosome
Ribosome
Oxidative phosphorylation
Huntington's disease
Aminoacyl-tRNA biosynthesis
Cell cycle
Parkinson's disease
RNA degradation
DNA replication
Pyrimidine metabolism
Nucleotide excision repair
Proteasome
Alzheimer's disease
Purine metabolism
Ubiquitin mediated proteolysis
RNA polymerase
Systemic lupus erythematosus
Oocyte meiosis
Basal transcription factors
Progesterone-mediated oocyte maturation
Mismatch repair
Homologous recombination
Terpenoid backbone biosynthesis
p53 signaling pathway
Citrate cycle (TCA cycle)
Vibrio cholerae infection
mTOR signaling pathway
N-Glycan biosynthesis
Insulin signaling pathway
Protein export
Renal cell carcinoma
Selenoamino acid metabolism
Porphyrin and chlorophyll metabolism
Base excision repair
Acute myeloid leukemia
Valine, leucine and isoleucine biosynthesis
Cardiac muscle contraction
Chronic myeloid leukemia
Epithelial cell signaling in Helicobacter pylori infection
Pentose phosphate pathway
Pathways in cancer
One carbon pool by folate
Endocytosis
Lysine degradation
Pathogenic Escherichia coli infection
Glutathione metabolism
Glyoxylate and dicarboxylate metabolism
Neurotrophin signaling pathway
Amino sugar and nucleotide sugar metabolism
Glycolysis / Gluconeogenesis
TGF-beta signaling pathway
ErbB signaling pathway
0 20 40 60 80 100
-log(FDR)
b
Vector: pKLV2-sgBFP-mCherry-2A-BFP
Vector: pKLV2-sgBFP-GFP-2A-BFP
MOLM-13
MOLM-13
THP1
HL60
Control
Cas9-expressing
BFP
mCherry
GFP
c
Relative to day 4 (%)
THP1
HL60
100 50 0
4 7 10 12 14 17
ADSL_sg1 ADSL_sg2 ADSS_sg1 ADSS_sg2 ATIC_sg1 ATIC_sg2 GART_sg1 GART_sg2 GMPS_sg1 GMPS_sg2 IMPDH2_sg1 IMPDH2_sg2 PAICS_sg1 PAICS_sg2 PFAS_sg1 PFAS_sg2 PPAT_sg1 PPAT_sg2 MYC_sg1 MYC_sg2 NC_sg
MOLM-13
THP1
HL60
NC_sg sg1 sg2
62
50
50
35
IMPDH2
β-actin
d
LSCs
MMF (IC50: 0.2447 μM)
MPA (IC50: 0.3906 μM)
6-MP (IC50: 4.495 μM)
Relative viability (%)
120 80 40 0
-2 -1 0 1
Log10 (μM)
e
MMF (μM) 0 0 0.25 0.5 0.75 1.0
SYTOX Deep Red labeled COH1 - + + + + +
Day 1
Day 2
R660 - SYTOX Deep Red
B530
f
Day 1
Day 2
Phagocyted cells (%)
100 80 60 40 20 0
0.021
<0.001
<0.001
<0.001
0.005
0.001
<0.001
0 0.25 0.50 0.75 1.00
MMF (μM)

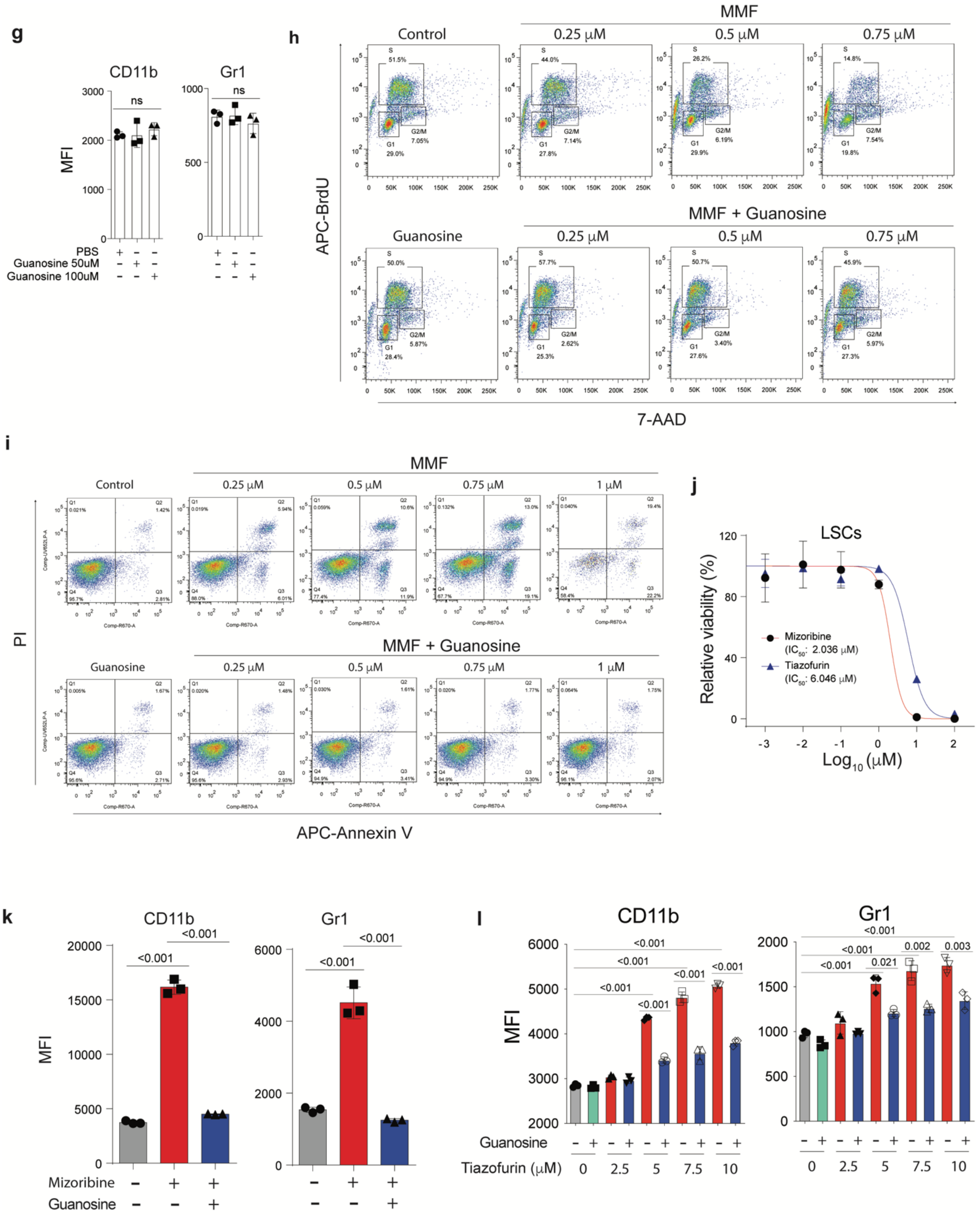
g
CD11b
Gr1
ns
ns
MFI
3000
2000
1000
0
1000
500
0
PBS + − −
Guanosine 50uM − + −
Guanosine 100uM − − +
h
Control
MMF
0.25 μM
0.5 μM
0.75 μM
S 51.5%
G1 29.0%
G2/M 7.05%
S 44.0%
G1 27.8%
G2/M 7.14%
S 26.2%
G1 29.9%
G2/M 6.19%
S 14.8%
G1 19.8%
G2/M 7.54%
APC-BrdU
Guanosine
MMF + Guanosine
0.25 μM
0.5 μM
0.75 μM
S 50.0%
G1 28.4%
G2/M 5.87%
S 57.7%
G1 25.3%
G2/M 2.62%
S 50.7%
G1 27.6%
G2/M 3.40%
S 45.9%
G1 27.3%
G2/M 5.97%
0 50K 100K 150K 200K 250K
7-AAD
i
Control
MMF
0.25 μM
0.5 μM
0.75 μM
1 μM
PI
Guanosine
MMF + Guanosine
0.25 μM
0.5 μM
0.75 μM
1 μM
APC-Annexin V
j
LSCs
Relative viability (%)
120
80
40
0
Mizoribine (IC50: 2.036 μM)
Tiazofurin (IC50: 6.046 μM)
-3 -2 -1 0 1 2
Log10 (μM)
k
CD11b
Gr1
<0.001
<0.001
<0.001
<0.001
MFI
20000
15000
10000
5000
0
6000
4000
2000
0
Mizoribine − + +
Guanosine − − +
l
CD11b
Gr1
MFI
6000
5000
4000
3000
2000
2000
1500
1000
500
0
<0.001
<0.001
<0.001
<0.001
<0.001
<0.001
<0.001
<0.001
<0.001
0.021
0.002
0.003
Guanosine − + − + − + − + − +
Tiazofurin (μM) 0 2.5 5 7.5 10

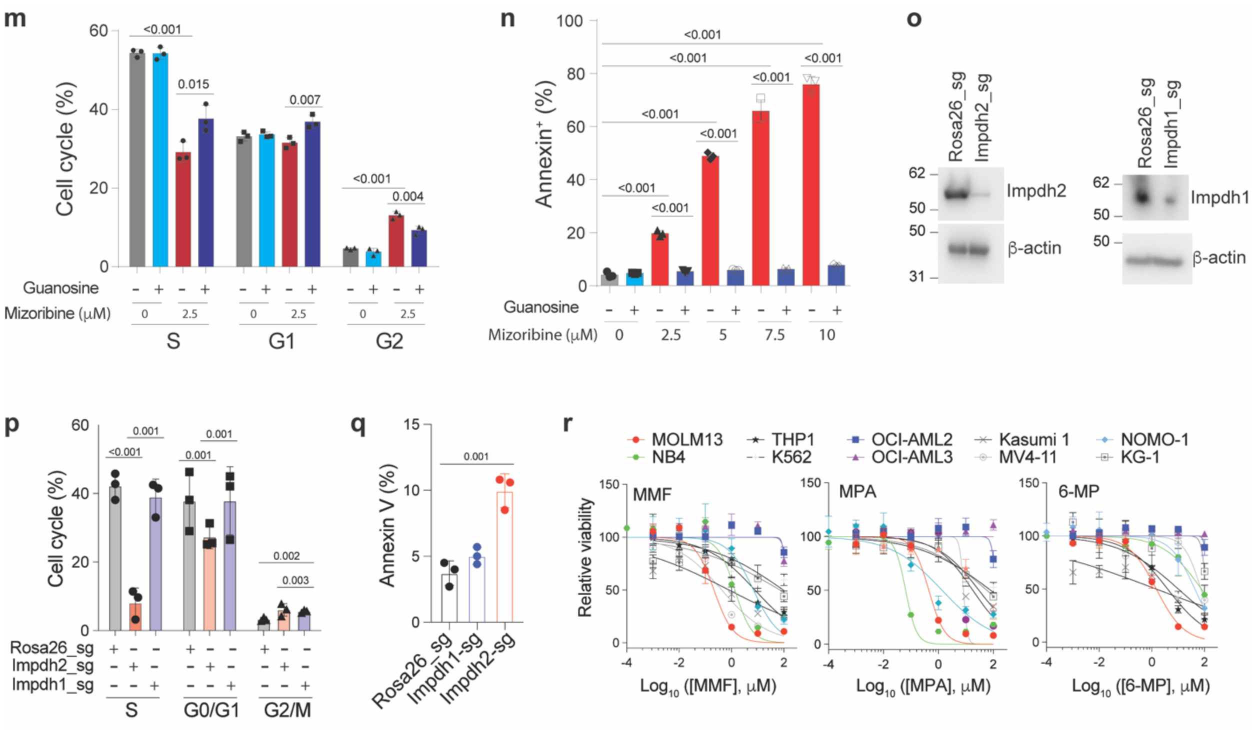

s

	IC_{50} (μM)			
	MMF	MPA	6-MP	MLL fusion
MOLM13	0.214	0.4501	1.483	MLL-AF9
THP1	7.823	30.1	3.979	MLL-AF9
MV4-11	0.425	8.201	73.38	MLL-AF4
NOMO1	6.891	1.371	43.2	MLL-AF9
NB4	1.342	0.0643	76.54	None
K562	94.73	80.73	10.14	None
Kasumi1	0.8013	18.08	1.47	None
KG1	70.97	58.3	115.4	None
OCI-AML2	140.4	126.4	129.3	MLL-AF6
OCI-AML3	115.5	n.a	n.a	None

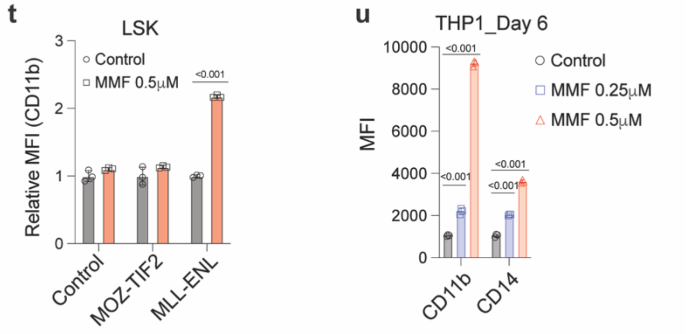

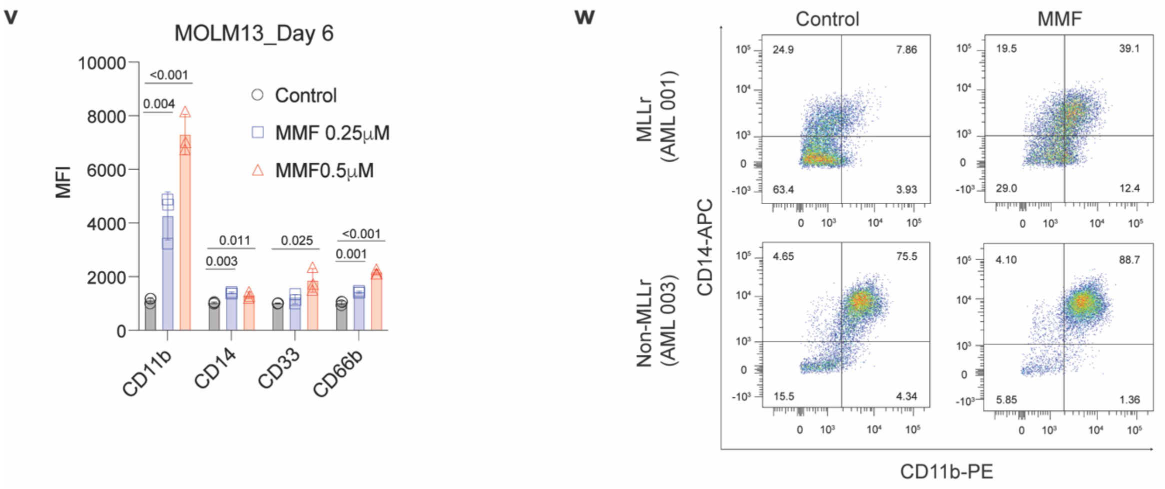

Supplementary Fig. 3. Inhibition of the purine biosynthetic pathway promotes myeloid differentiation of AML cells, related to Fig. 3.

(**a**) Gene ontology (GO) terms discovered from the dropout genes in a whole-genome CRISPR screen in MOLM-13 cells. A total of 52 gene sets were shown based on FDR < 0.01 and p < 0.01. Purine metabolism is indicated in red.

(**b**) Representative flow cytometry plots showing Cas9 function in MOLM-13, THP-1 and HL60 cells. Cells with (bottom panel) or without (upper panel) Cas9 expression were transduced with lentiviral vectors that express BFP and mCherry or GFP along with sgRNA against BFP. BFP expression becomes extinguished by the expression of sgBFP.

(**c**) Upper panel: Competitive growth assay in Cas9-expressing-THP-1 and HL-60 cells that express sgRNAs against negative control (NC), positive control (*MYC*), and genes involved in the purine biosynthetic pathway over 17 days. The percentages of sgRNA-expressing cells were normalized to those on day 4 after transduction. Bottom panel: Representative immunoblots of IMPDH2 and β-actin on separate membranes in Cas9-expressing MOLM-13, THP-1 and HL60 cells after the transduction of sgRNAs targeting *IMPDH2* (n=2).

(**d**) Dose-response curves of LSC viability with MMF, MPA, or 6-MP treatment for 2 days (n=3).

(**e-f**) Representative flow cytometry dot plots (**e**) and frequencies (**f**) of phagocytic activity of LSCs treated with MMF (0.25-1 μM) co-cultured with labeled Streptococcus agalactiae COH1 (n=3).

(**g**) Mean fluorescent intensity (MFI) of myeloid differentiation marker CD11b and Gr-1 in LSCs with guanosine (50-100 μM) treatment for 2 days (n=3). ns, not significant.

(**h-i**) Representative flow cytometry plots of BrdU incorporation (**h**) and apoptosis assay (**i**) of LSCs with MMF treatment (0.25-0.75 μM) for 24 hours.

(**j**) Dose-response curves for LSC cell viability with Tiazofurin or Mizoribine treatment for 2 days (n=3).

(**k-l**) MFI of myeloid differentiation marker CD11b (left panel) and Gr-1 (right panel) in LSCs treated with Mizoribine (**k**, 2 μM) or Tiazofurin (**l**, 2.5-10 μM) alone or in combination with guanosine (100 μM) (n=3).

(**m-n**) Cell cycle (**m**) and apoptosis (**n**) analysis of LSCs upon treatment with Mizoribine alone (2.5-10 μM) or in combination with guanosine (100 μM) (n=3).

(**o-q**) Immunoblots of IMPDH2, IMPDH1, and β-actin on separate membranes (**o**), cell cycle (**p**), and apoptosis (**q**) analysis in Cas9-expressing LSCs after the transduction of sgRNAs targeting *Imdph2* or *Impdh1* (n=3).

(**r-s**) Dose-response curves for cell viability (**r**) and IC_{50} (**s**) of a panel of AML cell lines treated with MMF, MPA, or 6-MP treatment for 3 days.

(**t**) Relative MFI of myeloid differentiation marker CD11b in LSK cells expressing vector control, MOZ-TIF2 or MLL-ENL, treated with MMF (0.5 μM) for 24 hours (n=3).

(**u-v**) MFI of the indicated myeloid differentiation markers in THP-1 (**u**) and MOLM-13 (**v**) cells treated with MMF (0.25-0.5 μM) for 6 days (n=3).

(**w**) Representative flow cytometry plots of myeloid differentiation CD11b and CD14 in *MLL*r and non-*MLL*r AML samples treated with MMF (1 μM) for 6 days.

All data are presented as mean ± SD. p values in this figure were calculated by unpaired, two-tailed Student's t-test (**t**) or ANOVA with multiple comparisons analysis using Dunnett's (**f, g, q, u, v**) or Bonferroni correction (**k-n, p**) post hoc analyses. Source data are provided as a Source Data file.

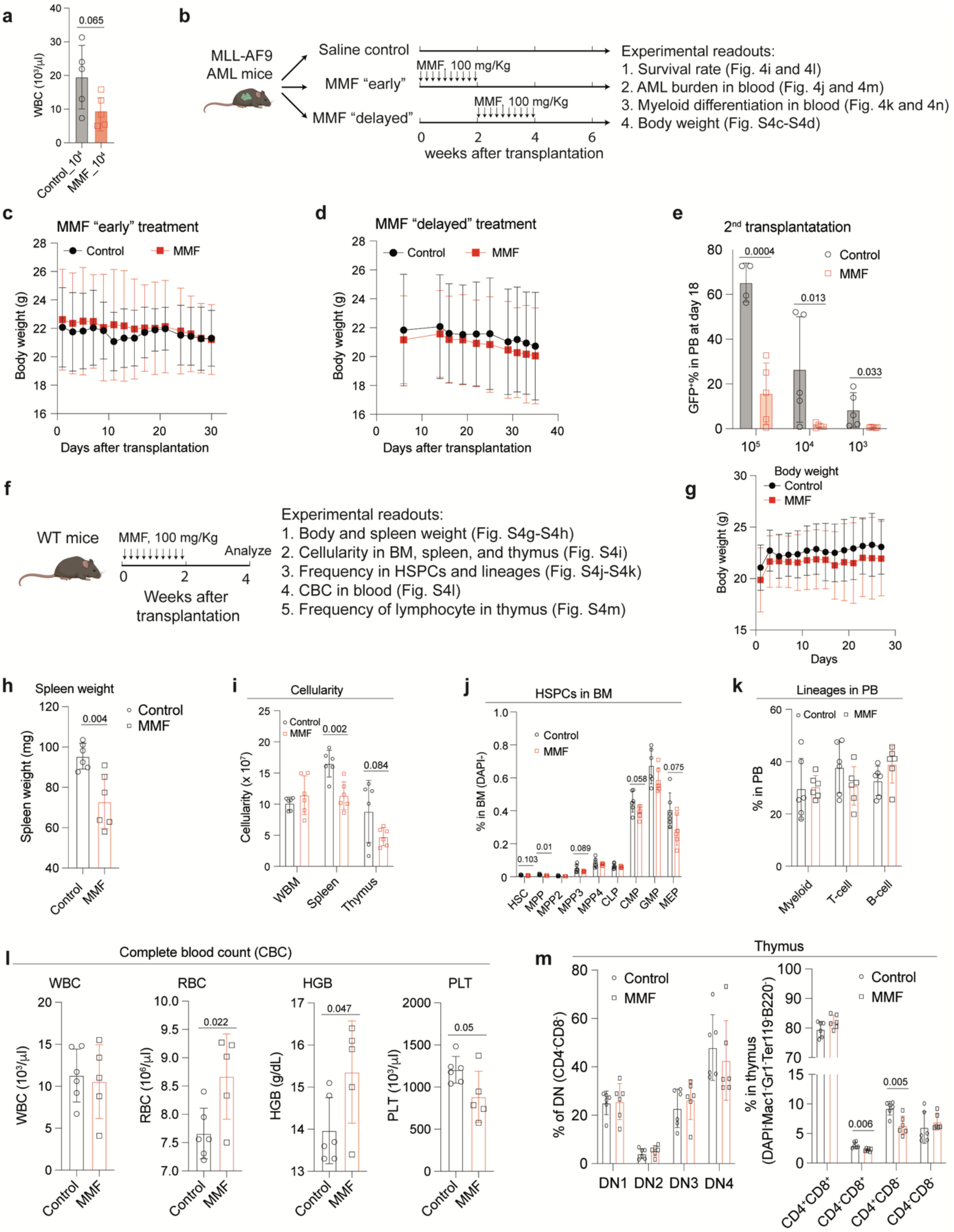
a
WBC (10³/µl)
0.065
Control_10⁴
MMF_10⁴
b
MLL-AF9
AML mice
Saline control
MMF "early"
MMF "delayed"
MMF, 100 mg/Kg
weeks after transplantation
Experimental readouts:
1. Survival rate (Fig. 4i and 4l)
2. AML burden in blood (Fig. 4j and 4m)
3. Myeloid differentiation in blood (Fig. 4k and 4n)
4. Body weight (Fig. S4c-S4d)
c
MMF "early" treatment
Control
MMF
Body weight (g)
Days after transplantation
d
MMF "delayed" treatment
Control
MMF
Body weight (g)
Days after transplantation
e
2nd transplantatation
GFP⁺% in PB at day 18
0.0004
0.013
0.033
Control
MMF
10⁵
10⁴
10³
f
WT mice
MMF, 100 mg/Kg
Analyze
Weeks after
transplantation
Experimental readouts:
1. Body and spleen weight (Fig. S4g-S4h)
2. Cellularity in BM, spleen, and thymus (Fig. S4i)
3. Frequency in HSPCs and lineages (Fig. S4j-S4k)
4. CBC in blood (Fig. S4l)
5. Frequency of lymphocyte in thymus (Fig. S4m)
g
Body weight
Control
MMF
Body weight (g)
Days
h
Spleen weight
Control
MMF
Spleen weight (mg)
0.004
i
Cellularity
Control
MMF
Cellularity (x 10⁷)
0.002
0.084
WBM
Spleen
Thymus
j
HSPCs in BM
Control
MMF
% in BM (DAPI-)
0.103
0.01
0.089
0.058
0.075
HSC
MPP
MPP2
MPP3
MPP4
CLP
CMP
GMP
MEP
k
Lineages in PB
Control
MMF
% in PB
Myeloid
T-cell
B-cell
l
Complete blood count (CBC)
WBC
RBC
HGB
PLT
WBC (10³/µl)
RBC (10⁶/µl)
HGB (g/dL)
PLT (10³/µl)
0.022
0.047
0.05
Control
MMF
m
Thymus
Control
MMF
% of DN (CD4⁻CD8⁻)
DN1
DN2
DN3
DN4
% in thymus
(DAPI⁻Mac1⁻Gr1⁻Ter119⁻B220⁻)
0.006
0.005
CD4⁺CD8⁺
CD4⁻CD8⁺
CD4⁺CD8⁻
CD4⁻CD8⁻

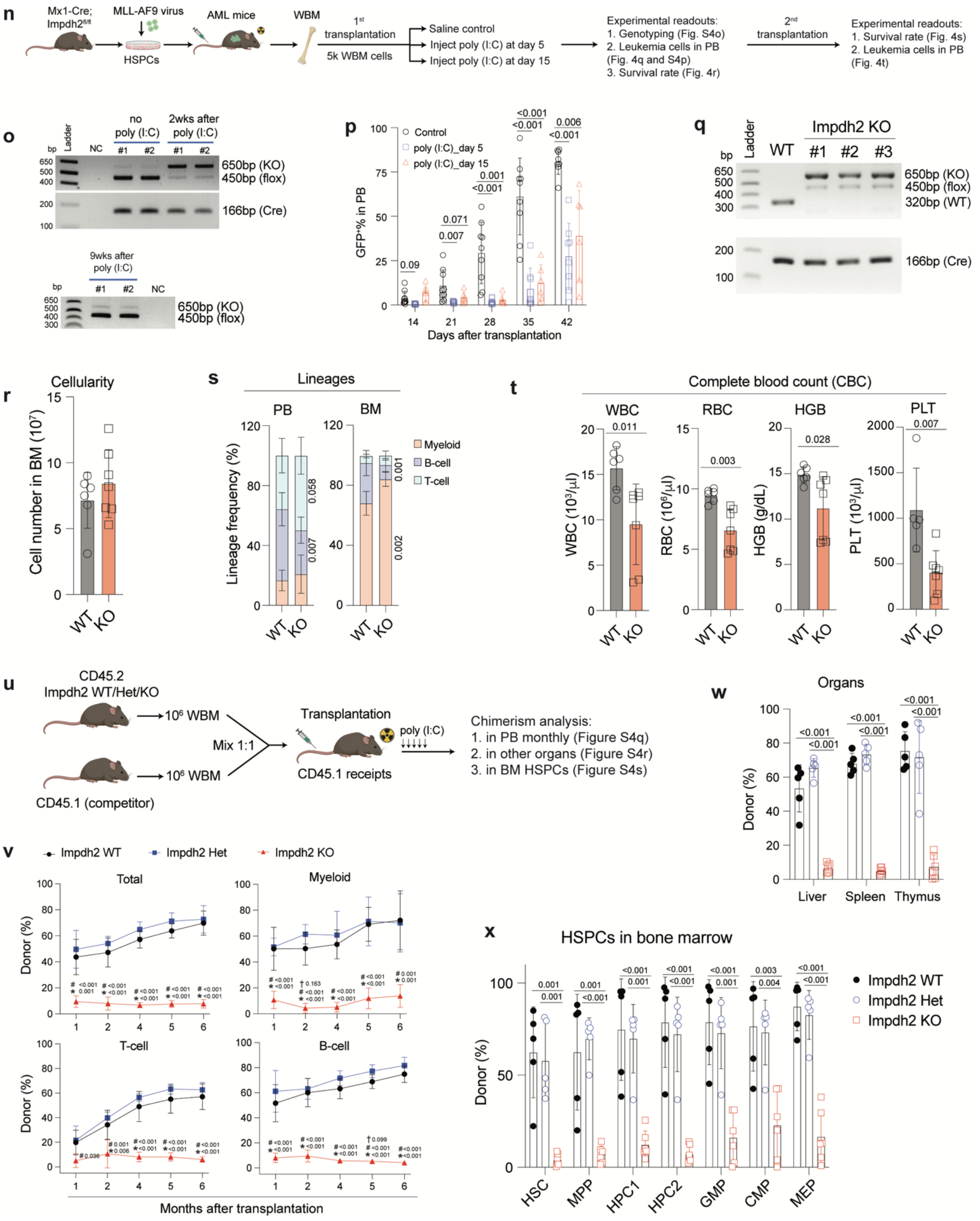
n
Mx1-Cre; Impdh2fl/fl
MLL-AF9 virus
HSPCs
AML mice
WBM
1st transplantation
5k WBM cells
Saline control
Inject poly (I:C) at day 5
Inject poly (I:C) at day 15
Experimental readouts:
1. Genotyping (Fig. S4o)
2. Leukemia cells in PB (Fig. 4q and S4p)
3. Survival rate (Fig. 4r)
2nd transplantation
Experimental readouts:
1. Survival rate (Fig. 4s)
2. Leukemia cells in PB (Fig. 4t)
o
Ladder
NC
no poly (I:C)
2wks after poly (I:C)
650bp (KO)
450bp (flox)
166bp (Cre)
9wks after poly (I:C)
p
Control
poly (I:C)_day 5
poly (I:C)_day 15
GFP+% in PB
Days after transplantation
q
Impdh2 KO
WT
650bp (KO)
450bp (flox)
320bp (WT)
166bp (Cre)
r
Cellularity
Cell number in BM (10^7)
s
Lineages
PB
BM
Lineage frequency (%)
Myeloid
B-cell
T-cell
t
Complete blood count (CBC)
WBC
RBC
HGB
PLT
WBC (10^3/μl)
RBC (10^6/μl)
HGB (g/dL)
PLT (10^3/μl)
u
CD45.2
Impdh2 WT/Het/KO
10^6 WBM
Mix 1:1
CD45.1 (competitor)
Transplantation
poly (I:C)
CD45.1 receipts
Chimerism analysis:
1. in PB monthly (Figure S4q)
2. in other organs (Figure S4r)
3. in BM HSPCs (Figure S4s)
v
Impdh2 WT
Impdh2 Het
Impdh2 KO
Total
Myeloid
T-cell
B-cell
Donor (%)
Months after transplantation
w
Organs
Donor (%)
Liver
Spleen
Thymus
x
HSPCs in bone marrow
Donor (%)
HSC
MPP
HPC1
HPC2
GMP
CMP
MEP
Impdh2 WT
Impdh2 Het
Impdh2 KO

Supplementary Fig. 4. The effects of purine biosynthesis inhibition on AML and normal hematopoiesis, related to Fig. 4.

(**a**) White blood cell (WBC) counts in PB of AML mice transplanted with 10^4 LSCs pretreated with PBS (control) or MMF (0.125 μM) *in vitro* for 12 hours (n=5).

(**b**) Experimental scheme for subjecting MLL-AF9 AML mice with "early" or "delayed" MMF treatment and the following analyses. Created in BioRender. Huang, S. (2025) https://BioRender.com/c04z111

(**c-d**) Body weight of AML mice treated with saline control, MMF "early", or "delayed" treatment (n=7-8).

(**e**) Frequency of GFP^+ AML cells in PB of secondary recipient mice transplanted with AML cells from mice that were exposed to MMF "early" treatment (n=5).

(**f**) Experimental scheme for subjecting C57BL/6 mice and the following analyses. Created in BioRender. Huang, S. (2025) https://BioRender.com/j05h386

(**g-m**) Body weight (**g**), spleen weight (**h**), cellularity (**i**), frequency of HSPCs in bone marrow (**j**), PB lineages (**k**), PB complete blood counts (CBC, **l**), and percentage of thymic cells (**m**) of C57BL/6 mice treated with or without MMF (n=6).

(**n**) Experimental scheme for generating a conditional Impdh2 knockout MLL-AF9 AML mice. Created in BioRender. Huang, S. (2025) https://BioRender.com/b92i975

(**o**) Genotyping of *Impdh2* and *Cre* alleles in MLL-AF9 AML cells after 2 (upper) or 9 weeks (bottom) of poly (I:C) injection. NC, negative control.

(**p**) Frequency of GFP^+ AML cells in PB of MLL-AF9-induced *Mx1-Cre; Impdh2*$^{fl/fl}$ AML mice with poly(I:C) injection at days 5 or 15 after transplantation.

(**q**) Genotyping of *Impdh2* and *Cre* alleles in whole bone marrow cells of *Mx1-Cre; Impdh2*$^{fl/fl}$ mice 3-4 months after poly (I:C) injection.

(**r-t**) Cellularity (**r**), lineage frequency in PB and bone marrow (**s**) and PB CBC (**t**) of *Impdh2* WT and KO mice (n=6-7).

(**u**) Schematic flowchart depicting competitive BM transplantation with *Impdh2* WT, Het, or KO WBM cells ($CD45.2^+$), along with competitor BM cells ($CD45.1^+$) into lethally irradiated $CD45.1^+$ recipient mice. Created in BioRender. Huang, S. (2025) https://BioRender.com/y85r771

(**v**) Percentage of overall donor cells (total), myeloid, T-cell, and B-cell in the recipient mice in experiments shown in Figure S4U. * compares Impdh2 WT vs KO, † compares WT vs Het, and # compares Het vs KO mice (n=5).

(**w-x**) Percentage of donor cells ($CD45.2^+$) in different organs (**w**) and HSPCs (**x**) in the bone marrow of the recipient mice 6 months after transplantation (n=5).

All data are presented as mean ± SD. p values in this figure were calculated by unpaired, two-tailed Student's t-test (**a, c-e, g-m, r-t**) or ANOVA with multiple comparisons analysis using Dunnett's (**p**) or Bonferroni correction (**v-x**) post hoc analyses. Source data are provided as a Source Data file.

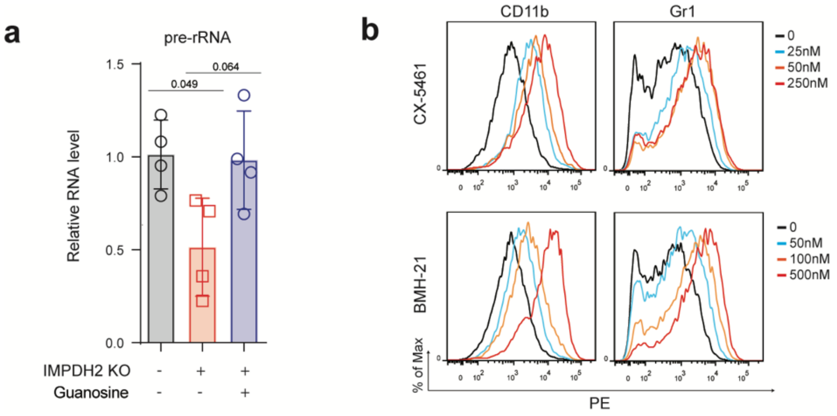

Supplementary Fig. 5. Disruption of rRNA transcription drives myeloid differentiation of LSCs *in vitro*, related to Fig. 5.

(**a**) Relative expression of pre-rRNA in MOLM-13 cells with or without *IMPDH2* deletion treated with guanosine (100 μM) for 24 hours (n=4).

(**b**) Flow cytometry histograms for mature myeloid cell markers CD11b (left panels) and Gr-1 (right panels) in LSCs upon treatment with control, CX-5461 (25-250 nM) or BMH-21 (50-500 nM) *in vitro* for 24 hours.

All data are presented as mean ± SD. p values in this figure were calculated by ANOVA with multiple comparisons analysis using Bonferroni correction post hoc analyses (**a**).

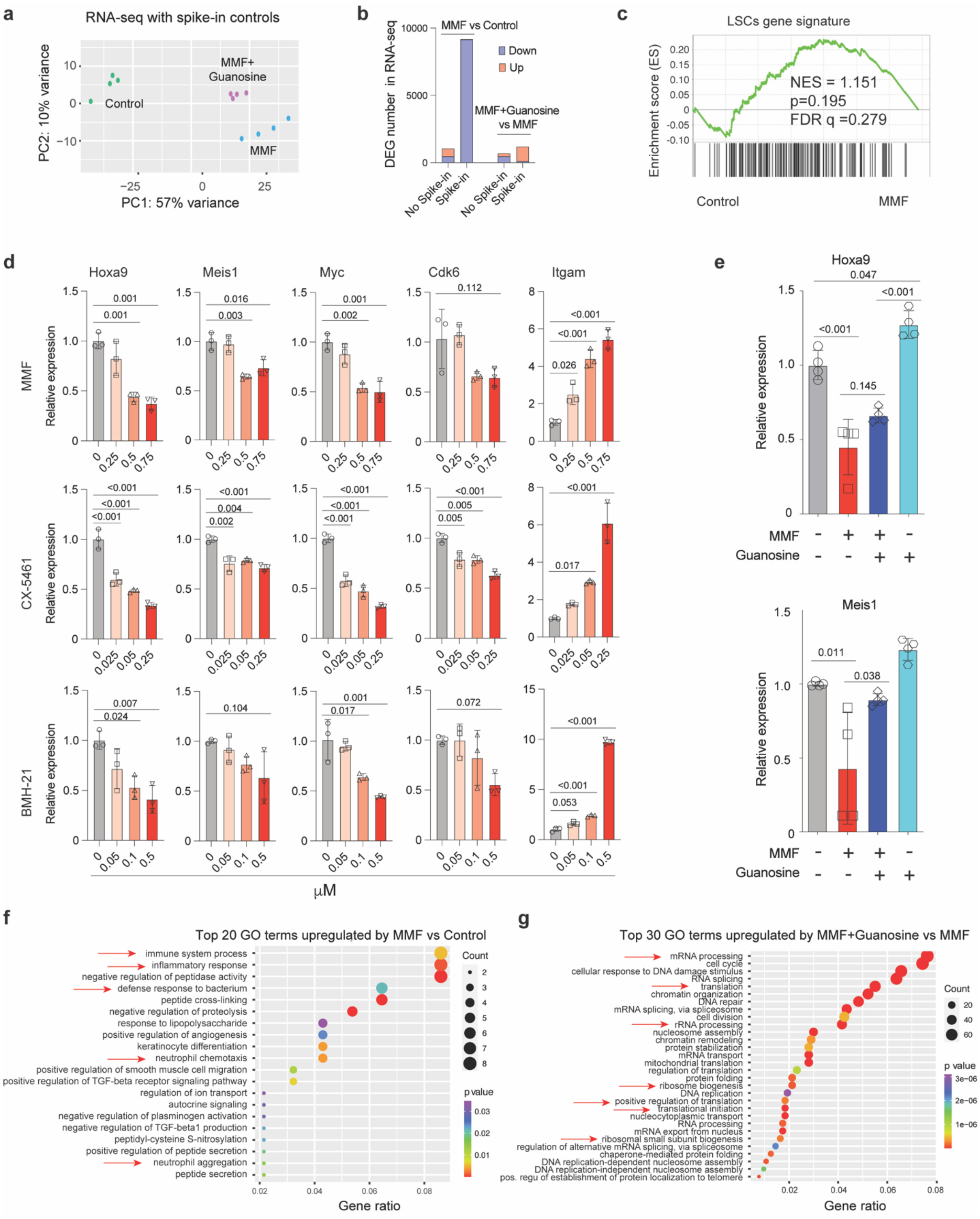
a
RNA-seq with spike-in controls
PC2: 10% variance
PC1: 57% variance
Control
MMF+
Guanosine
MMF
b
DEG number in RNA-seq
MMF vs Control
MMF+Guanosine
vs MMF
Down
Up
No Spike-in
Spike-in
c
LSCs gene signature
Enrichment score (ES)
NES = 1.151
p=0.195
FDR q =0.279
Control
MMF
d
Hoxa9
Meis1
Myc
Cdk6
Itgam
MMF
CX-5461
BMH-21
Relative expression
μM
e
Hoxa9
Meis1
Relative expression
MMF
Guanosine
f
Top 20 GO terms upregulated by MMF vs Control
immune system process
inflammatory response
negative regulation of peptidase activity
defense response to bacterium
peptide cross-linking
negative regulation of proteolysis
response to lipopolysaccharide
positive regulation of angiogenesis
keratinocyte differentiation
neutrophil chemotaxis
positive regulation of smooth muscle cell migration
positive regulation of TGF-beta receptor signaling pathway
regulation of ion transport
autocrine signaling
negative regulation of plasminogen activation
negative regulation of TGF-beta1 production
peptidyl-cysteine S-nitrosylation
positive regulation of peptide secretion
neutrophil aggregation
peptide secretion
Count
p value
Gene ratio
g
Top 30 GO terms upregulated by MMF+Guanosine vs MMF
mRNA processing
cell cycle
cellular response to DNA damage stimulus
RNA splicing
translation
chromatin organization
DNA repair
mRNA splicing, via spliceosome
cell division
rRNA processing
nucleosome assembly
chromatin remodeling
protein stabilization
mRNA transport
mitochondrial translation
regulation of translation
protein folding
ribosome biogenesis
DNA replication
positive regulation of translation
translational initiation
nucleocytoplasmic transport
RNA processing
mRNA export from nucleus
ribosomal small subunit biogenesis
regulation of alternative mRNA splicing, via spliceosome
chaperone-mediated protein folding
DNA replication-dependent nucleosome assembly
DNA replication-independent nucleosome assembly
pos. regu of establishment of protein localization to telomere
Count
p value
Gene ratio

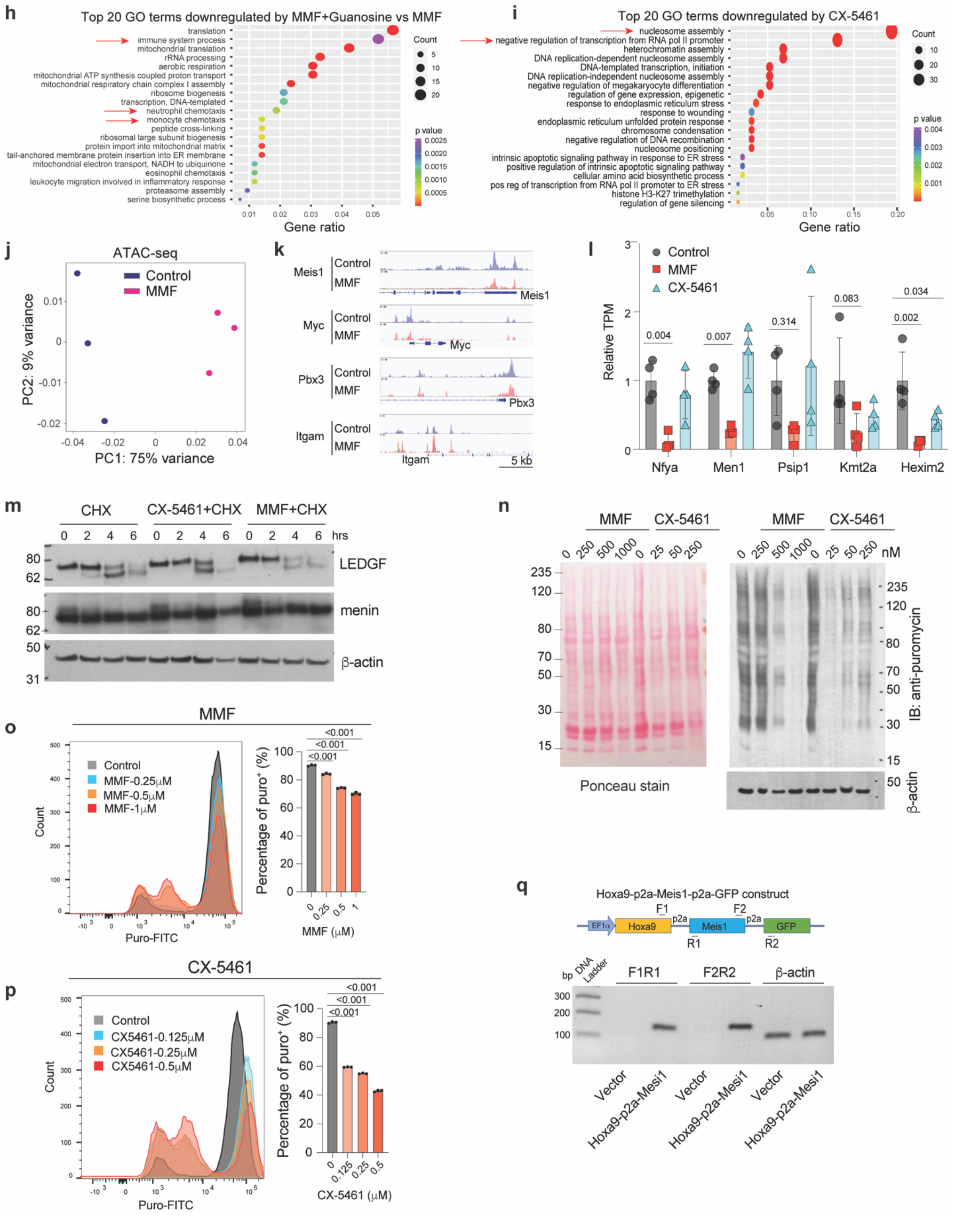
h
Top 20 GO terms downregulated by MMF+Guanosine vs MMF
translation
immune system process
mitochondrial translation
rRNA processing
aerobic respiration
mitochondrial ATP synthesis coupled proton transport
mitochondrial respiratory chain complex I assembly
ribosome biogenesis
transcription, DNA-templated
neutrophil chemotaxis
monocyte chemotaxis
peptide cross-linking
ribosomal large subunit biogenesis
protein import into mitochondrial matrix
tail-anchored membrane protein insertion into ER membrane
mitochondrial electron transport, NADH to ubiquinone
eosinophil chemotaxis
leukocyte migration involved in inflammatory response
proteasome assembly
serine biosynthetic process
Gene ratio
Count
p value
i
Top 20 GO terms downregulated by CX-5461
nucleosome assembly
negative regulation of transcription from RNA pol II promoter
heterochromatin assembly
DNA replication-dependent nucleosome assembly
DNA-templated transcription, initiation
DNA replication-independent nucleosome assembly
negative regulation of megakaryocyte differentiation
regulation of gene expression, epigenetic
response to endoplasmic reticulum stress
response to wounding
endoplasmic reticulum unfolded protein response
chromosome condensation
negative regulation of DNA recombination
nucleosome positioning
intrinsic apoptotic signaling pathway in response to ER stress
positive regulation of intrinsic apoptotic signaling pathway
cellular amino acid biosynthetic process
pos reg of transcription from RNA pol II promoter to ER stress
histone H3-K27 trimethylation
regulation of gene silencing
Gene ratio
Count
p value
j
ATAC-seq
Control
MMF
PC1: 75% variance
PC2: 9% variance
k
Meis1
Myc
Pbx3
Itgam
Control
MMF
5 kb
l
Control
MMF
CX-5461
Relative TPM
0.004
0.007
0.314
0.083
0.034
0.002
Nfya
Men1
Psip1
Kmt2a
Hexim2
m
CHX
CX-5461+CHX
MMF+CHX
0 2 4 6 0 2 4 6 0 2 4 6 hrs
LEDGF
menin
β-actin
n
MMF
CX-5461
0 250 500 1000 0 25 50 250 nM
Ponceau stain
IB: anti-puromycin
β-actin
o
MMF
Control
MMF-0.25μM
MMF-0.5μM
MMF-1μM
Count
Puro-FITC
Percentage of puro⁺ (%)
<0.001
MMF (μM)
p
CX-5461
Control
CX5461-0.125μM
CX5461-0.25μM
CX5461-0.5μM
Count
Puro-FITC
Percentage of puro⁺ (%)
<0.001
CX-5461 (μM)
q
Hoxa9-p2a-Meis1-p2a-GFP construct
EF1α
Hoxa9
p2a
Meis1
GFP
F1
R1
F2
R2
bp
DNA Ladder
F1R1
F2R2
β-actin
Vector
Hoxa9-p2a-Mesi1

Supplementary Fig. 6. Inhibition of purine biosynthesis reduces LSCs gene expression, related to Fig. 6.

(**a**) Principal component analysis (PCA) plot of the RNA-seq transcriptome derived from LSCs treated with control, MMF, or MMF plus guanosine for 16 hours.

(**b**) Comparison of the number of Differentially Expressed Genes (DEG) identified from the RNA-seq data with or without spike-in control.

(**c**) Gene set enrichment analysis (GSEA) plots showing negative enrichment of the LSC gene set (PMID: 19200802) in MMF-treated versus control LSCs.

(**d**) Relative expression of *Hoxa9, Meis1, Myc, Cdk6* and *Itgam* in LSCs treated with or without MMF (0.25-0.75 μM), CX-5461(0.025-0.25 μM), or BMH-21 (0.05-0.5 μM) for 16 hours (n=3). ns, not significant.

(**e**) Relative expression of *Hoxa9* and *Meis1* in LSCs treated with control, MMF (0.5 μM), guanosine (100 μM), or MMF (0.5 μM) and guanosine (100 μM) for 16 hours (n=3).

(**f**) Top 20 Gene Ontology (GO) terms analyzed by DAVID for genes upregulated in LSCs by MMF compared to control. The terms highlighted with red arrows are associated with neutrophil activity.

(**g**) Top 30 GO terms analyzed by DAVID for genes upregulated by MMF and guanosine compared to MMF alone in LSCs. The terms highlighted with red arrows are associated with rRNA transcription and protein translation.

(**h**) Top 20 GO terms analyzed by DAVID for genes downregulated in LSCs by MMF and guanosine compared to MMF alone. The terms highlighted with red arrows are associated with neutrophil activity.

(**i**) Top 20 GO terms analyzed by DAVID for genes downregulated in LSCs by CX-5461 compared to control.

(**j**) PCA plot of ATAC-seq results from LSCs treated with or without MMF (0.25 μM) for 16 hours.

(**k**) ATAC-seq occupancy profiles from LSCs treated with or without MMF (0.25 μM) at the indicated locus.

(**l**) Relative transcripts per million (TPM) of *Nfya, Men1, Psip1, Kmt2a,* and *Hexim2* from RNA-seq data of LSCs treated with control, MMF (0.25 μM), or CX-5461 (0.1 μM) for 16 hours.

(**m**) Immunoblots of LEDGF, Menin, and β-actin on separate membranes in LSCs treated with CX-5461 (0.25 μM) or MMF (1 μM) in the presence of cycloheximide (CHX, 50 mg/ml) for the indicated time.

(**n**) Ponceau stain (left panel) and immunoblots of puromycin and β-actin (right panel) in LSCs treated with MMF (250-1000 nM) or CX-5461 (25-250 nM) for 16 hours.

(**o-p**) Flow cytometry histograms of puromycin incorporation (left panels) and percentage of the puromycin$^+$ (right panels) in LSCs treated with or without MMF (0.25-1 μM) (**o**) and CX-5461 (0.125-0.5 μM) (**p**) by flow cytometry.

(**q**) Schematic picture of Hoxa9-p2a-Meis1-p2a-GFP construct (upper panel) and detection of the construct alongside with β-actin (bottom panel), serving as an internal control, in THP1 cells overexpressed with either empty vector or the Hoxa9-p2a-Meis1-p2a-GFP construct.

All data are presented as mean ± SD. p values in this figure were calculated by ANOVA with multiple comparisons analysis using Dunnett's (**d, l, o, p**) or Bonferroni correction (**e**) post hoc analyses. Source data are provided as a Source Data file.

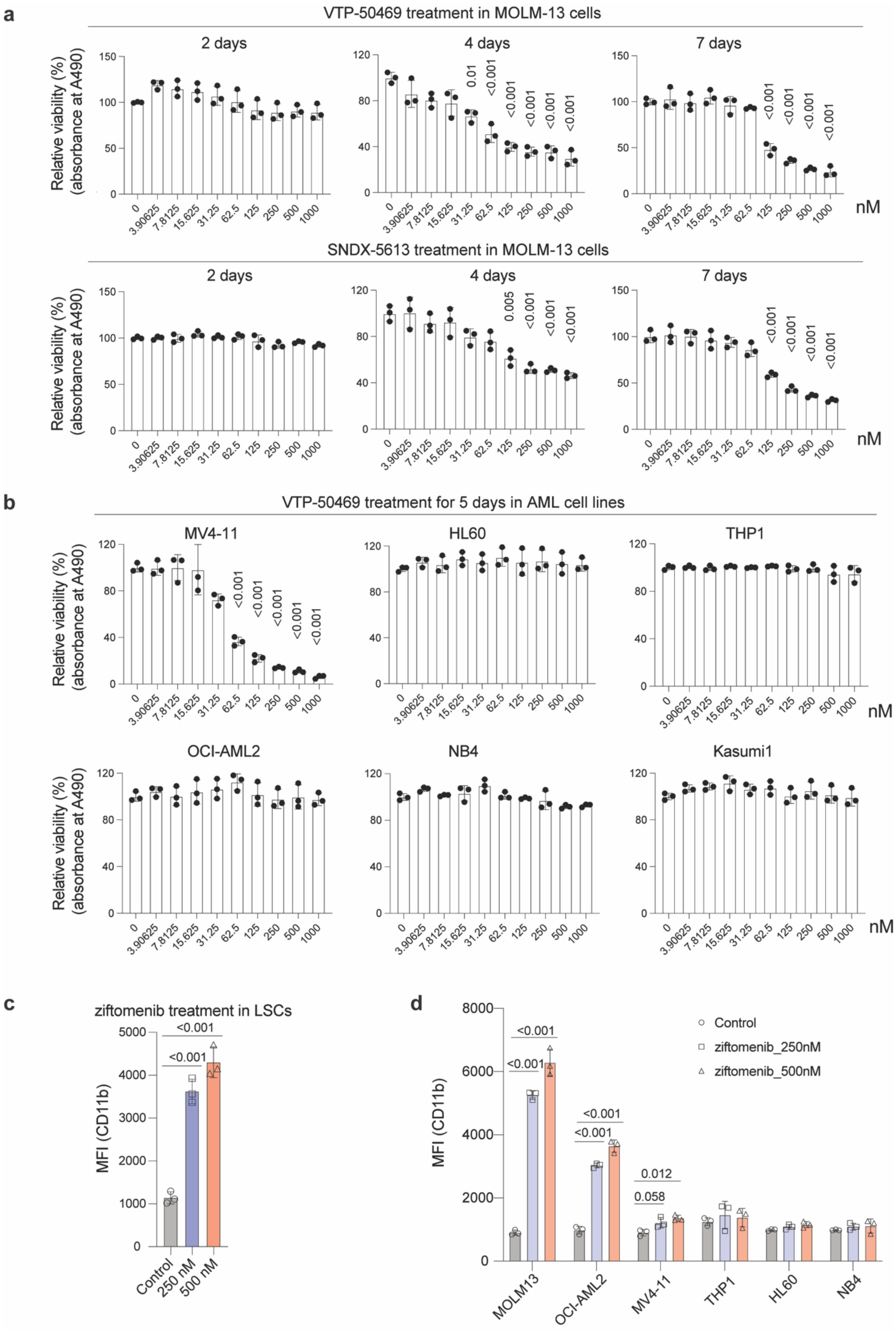
a
VTP-50469 treatment in MOLM-13 cells
2 days
4 days
7 days
Relative viability (%) (absorbance at A490)
0
3.90625
7.8125
15.625
31.25
62.5
125
250
500
1000
nM
0.01
<0.001
SNDX-5613 treatment in MOLM-13 cells
0.005
b
VTP-50469 treatment for 5 days in AML cell lines
MV4-11
HL60
THP1
OCI-AML2
NB4
Kasumi1
c
ziftomenib treatment in LSCs
MFI (CD11b)
Control
250 nM
500 nM
d
Control
ziftomenib_250nM
ziftomenib_500nM
0.012
0.058
MOLM13
OCI-AML2
MV4-11
THP1
HL60
NB4

e

Summary of mean Synergy Score for drug combinations							
Genetic alteration	Cell lines	Drug combinations	Experiments	ZIP	Loewe	HSA	Bliss
MLL translocation	MOLM-13	MMF & VTP-50469	1st	1.15	2.15	6.08	1.32
			2nd	2.11	4.05	6.67	2.3
			3rd	7.96	0.73	4.34	8.82
		CX-5461 & VTP-50469	1st	9.25	10.97	14.71	9.85
			2nd	7.71	5.76	14.61	7.81
			3rd	7.58	10.65	14.09	7.19
		MMF & Doxorubicin	1st	-1.42	-3.45	2.35	-1.54
			2nd	-2.04	-4.58	0.84	-2.17
			3rd	-4.58	-8.69	-2.9	-5.14
	MV4-11	MMF & VTP-50469	1st	1.51	5.07	10.53	1.18
			2nd	2.76	2.33	8.35	2.66
			3rd	3.66	5.3	10.21	3.72
		CX-5461 & VTP-50469	1st	2.99	8.36	12.98	3.06
			2nd	9.13	11.86	16.96	8.75
			3rd	8.26	10.94	16.16	8.03
		MMF & Doxorubicin	1st	3.11	-9.42	-3.51	4.08
			2nd	1.37	-5.1	-2.4	1.58
			3rd	1.81	-3.9	-0.62	1.7
Non-MLL translocation	NB4	MMF & VTP-50469	1st	4.45	1.87	1.77	4.5
			2nd	-1.25	0.02	-0.31	-1.69
			3rd	-0.17	0.65	0.36	-0.42
		CX-5461 & VTP-50469	1st	-6.37	-0.28	-0.61	-7.72
			2nd	-1.63	-1.06	-1.48	-2.46
			3rd	-0.74	-0.72	-0.7	-1.18

f

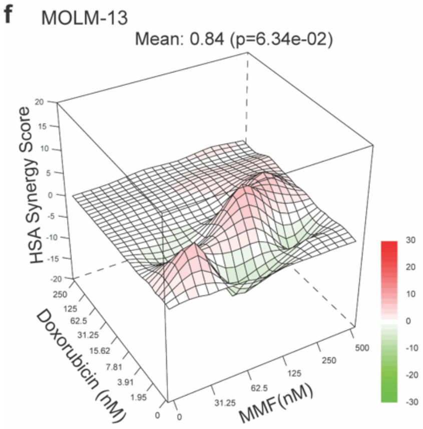

g

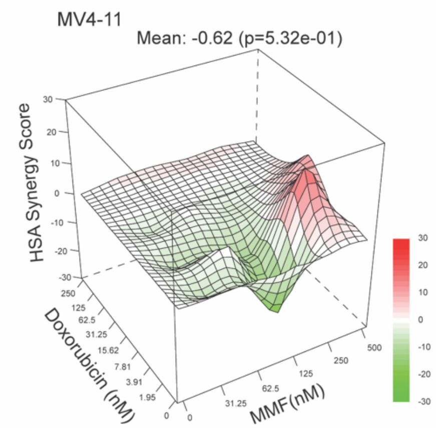

h

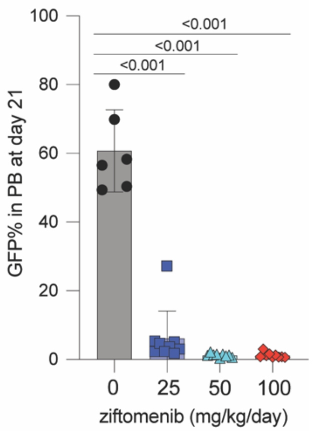

Supplementary Fig. 7. Response of Menin inhibitors on AML cells and leukemia mice, related to Fig. 7.

(**a**) Relative viability of MOLM-13 cells following treatment with different concentrations of VTP-50469 (3.91-1000 nM) and SNDX-5613 (3.91-1000 nM) over 2-7 days (n=3).

(**b**) Relative viability of human AML cell lines upon treatment with different concentrations of VTP-50469 (3.91-1000 nM) over 5 days (n=3).

(**c-d**) MFI of myeloid differentiation marker CD11b in LSCs (**c**) and human AML cell lines (**d**) treated with ziftomenib (250-500 nM) for 24 hours (**c**) and 4 days (**d**), respectively (n=3).

(**e**) Summary table of mean synergy score for drug combinations in AML cells using synergyfinder with ZIP, Loewe, HSA and Bliss models (n=3).

(**f-g**) HSA synergy score of MMF & Doxorubicin in MOLM-13 (**f**) and MV4-11 cells (**g**).

(**h**) Frequency of GFP^+ AML cells in PB of AML mice treated with control, ziftomenib (25-100 mg/kg/day) post 21 days of transplantation (n=6-9).

All data are presented as mean ± SD. p values in this figure were calculated by ANOVA with multiple comparisons analysis using Dunnett's post hoc analyses (**a-d, h**). Source data are provided as a Source Data file.

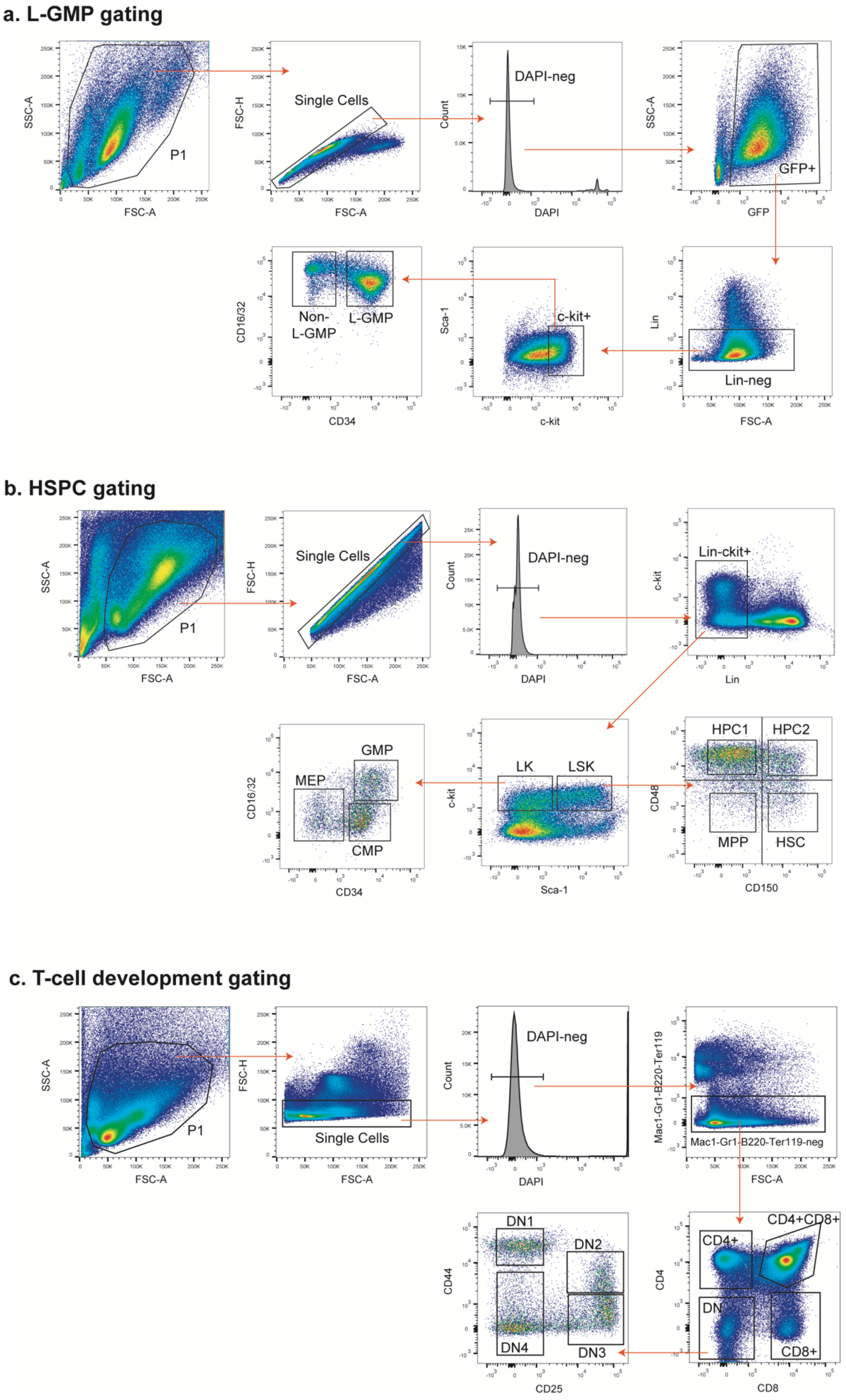
a. L-GMP gating
P1
SSC-A
FSC-A
Single Cells
FSC-H
DAPI-neg
Count
DAPI
GFP+
GFP
Lin-neg
Lin
c-kit+
Sca-1
c-kit
Non-
L-GMP
L-GMP
CD16/32
CD34
b. HSPC gating
P1
SSC-A
FSC-A
Single Cells
FSC-H
DAPI-neg
Count
DAPI
Lin-ckit+
c-kit
Lin
HPC1
HPC2
MPP
HSC
CD48
CD150
LK
LSK
Sca-1
GMP
MEP
CMP
CD16/32
CD34
c. T-cell development gating
P1
SSC-A
FSC-A
Single Cells
FSC-H
DAPI-neg
Count
DAPI
Mac1-Gr1-B220-Ter119
Mac1-Gr1-B220-Ter119-neg
CD4+CD8+
CD4+
DN
CD8+
CD4
CD8
DN1
DN2
DN3
DN4
CD44
CD25

d. lineage and competitive transplantation gating

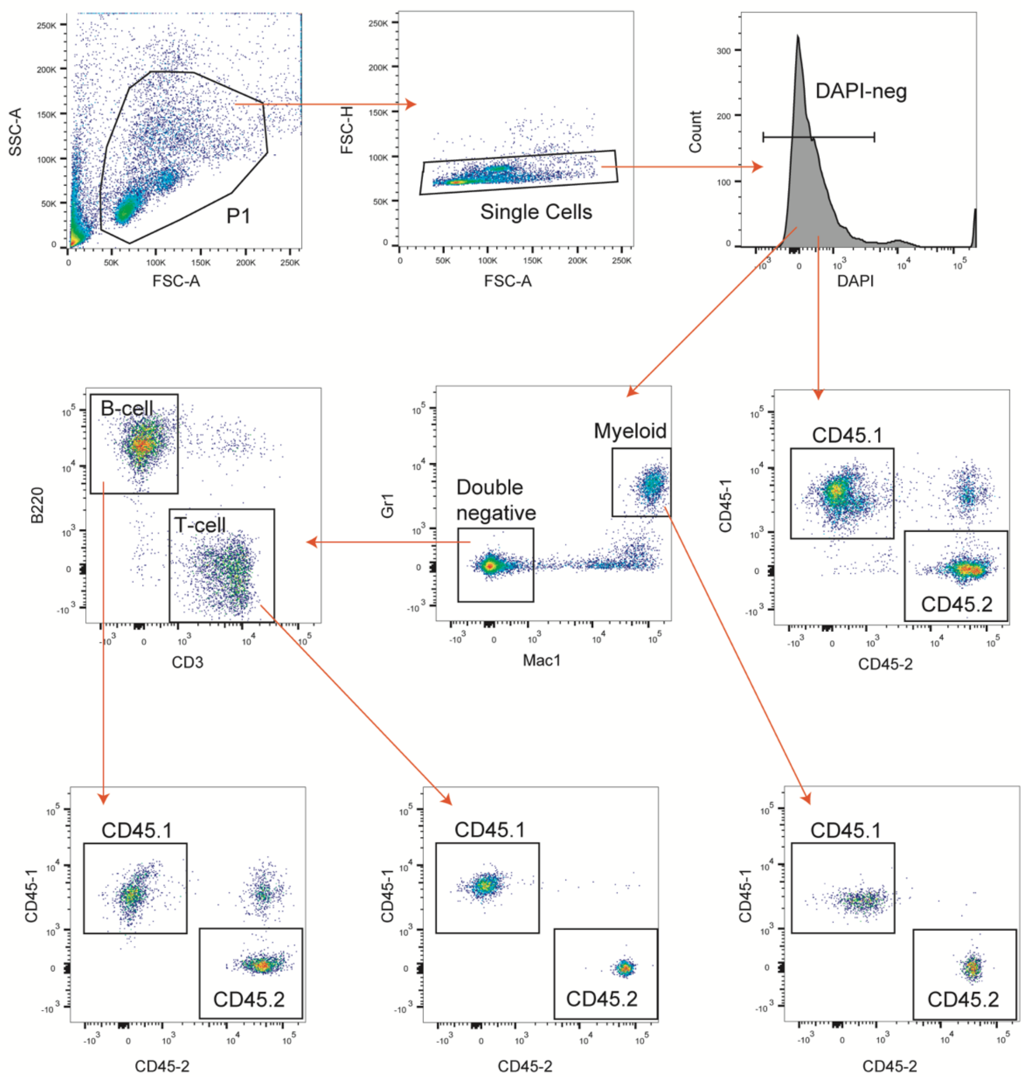

Supplementary Fig. 8. Representative flow cytometry gating strategy for L-GMP (**a**), HSPC (**b**), T-cell development (**c**), and lineage and competitive transplantation analysis (**d**). L-GMP, leukemia-GMP. DN, double negative.

Inventory of Supplemental Tables

Supplementary Data 1: Metabolomics profiles of LSCs, bulk AML, GMPs and WBM cells, related to Fig. 1.

Supplementary Data 2: Transcription factors binding to the promoter regions of purine biosynthesis genes from the ChIP-Atlas, related to Fig. 2.

Supplementary Data 3: Characteristics of AML samples used in the study, related to Fig. 3.

Supplementary Data 4: Differentially Expressed Genes (DEGs) from RNA-seq data of MMF vs Control, MMF+Guansine vs MMF, and CX-5461 vs Control, related to Fig. 6. Down, downregulated genes at adjusted $p < 0.05$ and log_2foldchange < -1; Up, upregulated genes at adjusted $p < 0.05$ and log_2foldchange > 1.

Supplementary Data 5: DEGs output of MMF vs Control RNA-seq data, related to Fig. 6.

Supplementary Data 6: DEGs output of MMF+Guansine vs MMF RNA-seq data, related to Fig. 6.

Supplementary Data 7: DEGs output of CX-5461 vs Control RNA-seq data, related to Fig. 6.

Supplementary Data 8: Primers used in the study.
