# Supplementary material for: Guanine nucleotide biosynthesis blockade impairs MLL complex formation and sensitizes leukemias to menin inhibition
Source: Nat Commun. 2025 Mar 18;16:2641. doi: 10.1038/s41467-025-57544-9 (PMC11920272; doi:10.1038/s41467-025-57544-9)
Supplement: Supplementary file 2 — Description of Additional Supplementary Information [file 41467_2025_57544_MOESM2_ESM.docx]

**Description of Additional Supplementary Files**

File Name: Supplementary Data 1

Description: Metabolomics profiles of LSCs, bulk AML, GMPs and WBM cells, related to Fig. 1.

File Name: Supplementary Data 2

Description: Transcription factors binding to the promoter regions of purine biosynthesis genes from the ChIP-Atlas, related to Fig. 2.

File Name: Supplementary Data 3

Description: Characteristics of AML samples used in the study, related to Fig. 3.

File Name: Supplementary Data 4

Description: Differentially Expressed Genes (DEGs) from RNA-seq data of MMF vs Control, MMF+Guansine vs MMF, and CX-5461 vs Control, related to Fig. 6. Down, downregulated genes at adjusted p < 0.05 and log2foldchange < -1; Up, upregulated genes at adjusted p < 0.05 and log2foldchange > 1.

File Name: Supplementary Data 5

Description: DEGs output of MMF vs Control RNA-seq data, related to Fig. 6.

File Name: Supplementary Data 6

Description: DEGs output of MMF+Guansine vs MMF RNA-seq data, related to Fig. 6.

File Name: Supplementary Data 7

Description: DEGs output of CX-5461 vs Control RNA-seq data, related to Fig. 6.

File Name: Supplementary Data 8

Description: Primers used in the study.
